# Reducing rural veteran suicides: Navigating geospatial and community contexts for scaling up a national Veterans Affairs program

**DOI:** 10.1111/sltb.12710

**Published:** 2021-04-20

**Authors:** Carlee J. Kreisel, Lauren K. Wilson, Alexandra L. Schneider, Nathaniel V. Mohatt, Talia L. Spark

**Affiliations:** ^1^ Rocky Mountain Mental Illness Research Education and Clinical Center (MIRECC) Rocky Mountain Regional VA Medical Center, U.S. Department of Veterans Affairs Aurora CO USA; ^2^ Department of Physical Medicine and Rehabilitation University of Colorado Anschutz Medical Campus Aurora CO USA; ^3^ GeoSpatial Outcomes Division (GSOD), Veterans Rural Health Resource Center (VRHRC‐GNV), VHA Office of Rural Health (ORH) U.S. Department of Veterans Affairs Gainesville FL USA; ^4^ Department of Psychiatry Yale University School of Medicine New Haven CT USA

## Abstract

**Objective:**

To develop and use planning maps to prioritize and facilitate county‐level recruitment for Together With Veterans (TWV), community‐based rural Veteran suicide prevention program.

**Method:**

Choropleth maps were created for 49 U.S. states, with four mutually exclusive categories indicating eligibility for the TWV program and increasing levels of need assigned to each county based on (a) percent Veterans Health Administration enrollees residing in rural communities, (b) percent population that are Veterans, and (c) crude suicide mortality rate.

**Results:**

Of 3113 counties, 78.2% were eligible for TWV and 25.8% met our highest priority definition. A national map and state map were provided to demonstrate final products used to engage stakeholders. A table of recommendations for creating and using planning maps was provided for future projects to reference.

**Conclusions:**

Geographic information system (GIS) is useful for identifying and prioritizing counties that may benefit most from a rural Veteran suicide prevention program. Choropleth maps allow for dissemination of information about county suicide risk and need for suicide prevention to community members, researchers, and others with a vested interest in suicide reduction. The maps are one tool among many which can support decision‐makers in focusing available resources on populations with the most need.

## 1 INTRODUCTION

Suicide rates have increased in the United States between 1999 and 2015 across all urban and rural levels, with rural areas having higher rates than urban areas and rates that are increasing more rapidly over time (Kegler et al., 2017). Veterans die by suicide at 1.5 times the rate of their non‐Veteran counterparts (U.S. Department of Veterans Affairs, 2019a) with rural Veterans at a 20%–22% greater risk of dying by suicide than urban Veterans (McCarthy et al., 2012).

To address this disparity, the Rocky Mountain Mental Illness Research, Education, and Clinical Center (MIRECC) developed, with funding from VA’s Office of Rural Health (ORH) and in partnership with the Office of Mental Health and Suicide Prevention (OMHSP), the Together With Veterans (TWV) community‐based suicide prevention program for rural Veterans (https://www.mirecc.va.gov/visn19/togetherwithveterans). In alignment with the VA National Strategy for Preventing Veteran Suicide 2018–2028, TWV supports the dissemination of evidence‐based best practices in public health suicide prevention (U.S. Department of Veterans Affairs, 2018) to reduce stigma and promote help‐seeking, promote lethal means safety, provide individual suicide prevention training, enhance primary care suicide prevention, and improve access to quality care (Mohatt et al., 2018; Monteith et al., 2020). TWV emphasizes Veteran leadership by supporting the development of a local, Veteran majority coalition to build community capacity through strategic planning, facilitation, and quality improvement support to implement evidence‐based suicide prevention strategies.

Utilizing county‐level data and geographic information system (GIS) to quickly visualize county suicide prevention need can be invaluable in facilitating conversations with stakeholders to narrow down a list of possible intervention communities, framing the conversation around community risk and need, not just community readiness (Lyseen et al., 2014; Nykiforuk & Flaman, 2011). Numerous studies have used GIS to address access to care issues for different patient populations, such as acute stroke, multiple sclerosis, and traumatic injuries (Cowper Ripley et al., 2019, 2017; Culpepper et al., 2010; Nykiforuk & Flaman, 2011; Ripley et al., 2009, 2014, 2015). Other work has described planning health services for populations of patients with specific medical conditions (Culpepper et al., 2010; Ripley et al., 2009, 2014, 2015). We discuss the process and utility of using GIS to visually represent and categorize U.S. counties by varying levels of need for the TWV program.

### 1.1 Background

A crucial step in the TWV process is working with Veterans Health Administration (VHA) officials and/or other state‐ or county‐level leadership to identify and recruit rural communities with high Veteran populations and increased suicide risk that would benefit most from the program. In the early stages of the Together With Veterans program, we sought high‐risk communities to pilot test the project. Our state stakeholders reported they wanted both to glean a quick picture of where the highest need was in their state and a data‐driven approach to expanding the program. With the early demonstration sites, we found that including a large number of data points in order to identify high‐risk communities introduced too much competing information to easily identify need for the program. In order to support stakeholders need for a simple tool, we chose to revise our approach to focus on a limited set of core variables. The TWV research team created maps in ArcGIS to prioritize counties with the highest level of need using three core county‐level variables: existing high rates of suicide, percent Veterans, and percent rural Veterans.

The planning maps discussed in this paper serve as a decision tool rather than a comprehensive review of health risk at the county level. The variables reflected in the maps are an intentionally simplified set of data to assist identification of sites where a rural Veteran suicide prevention program may be most impactful.

## 2 METHOD

### 2.1 Overview

Maps were created for a total of 49 states (Washington D.C. included in the Maryland map). Alaska was excluded for the current set of maps using county‐level data because the state does not have counties and multiple census boundaries changed as a result of the 2010 Census. Since our suicide data were aggregated from 2005 to 2017, it was not possible to include Alaska at this time (CDC, 2019d). Implications of this exclusion and future plans to address it are discussed later in the manuscript.

Need categories were created at the county level considering three factors: percent rural Veteran VHA enrollees, suicide rate, and percent of the population that are Veterans. County‐level data were used instead of state‐level data because (a) states contain a mix of rural and urban locations and this paper aims to identify rural places; (b) Veteran population and adult suicide rates vary substantially by geography within states, so providing state‐level summative data do not help identify locations of need within states, which is the goal of this work.

### 2.2 Data sources

The 2017 percentages of VHA enrollees residing in *rural* or *highly rural* designated census tracts of all VHA enrollees (which is aggregated up to the county) were sourced from the Current Enrollment Cube provided by the VHA Support Service Center (VSSC; Cowper Ripley et al., 2019, 2017). The VA uses Rural–Urban Commuting Area (RUCA) codes to define rurality, where *urban* areas are census tracts with 30 percent or more of residents living in an urbanized area per the Census Bureau (United States Department of Veterans Affairs, n.d.). *Highly Rural* refers to areas where <10% of working residents commute to areas larger than urbanized clusters (i.e., larger than micropolitan or small town cores), and r*ural* refers to any areas not defined as *urban* or *highly rural* (United States Department of Veterans Affairs, n.d.).

Veterans Affairs facility information was provided by the Planning Systems Support Group (PSSG) and categorized as “VA Medical Center” or “Community‐Based and Other Outpatient Clinics” (U.S. Department of Veterans Affairs, 2019b). PSSG stores information on the 1935 VA facilities, which is maintained regularly by VA network editors and administrations nationwide. VA Medical Centers provide numerous services such as surgery, critical care, mental health, radiology, and pharmacy, while Community‐Based and Other Outpatient Clinics (CBOCs) are smaller facilities and provide common outpatient services likes health and wellness visits (U.S. Department of Veterans Affairs, 2019c).

National county‐level suicide mortality rates for all adult residents in a county (Veteran and non‐Veteran) were provided by the VA Serious Mental Illness Treatment Resource and Evaluation Center (SMITREC). Original data were captured from the Centers for Disease Control and Prevention (CDC) Wide‐ranging Online Data for Epidemiologic Research (WONDER) Underlying Cause of Death database (CDC & NCHS, 2018). Crude county suicide rates per 100,000 residents were calculated using data from all adults (18+ years) who died by suicide (i.e., International Statistical Classification of Diseases, Tenth Revision (ICD‐10) underlying cause of death codes of X60–X84, Y87.0, *U03 (intentional self‐harm)) in the United States between 2005 and 2017 (CDC, 2019b). Due to confidentiality requirements, counties with less than ten suicide counts are suppressed in CDC WONDER (CDC, 2019a). CDC WONDER provides age‐adjusted suicide rates, but designates counties with 10–19 deaths as “unreliable” (CDC, 2019c). To minimize the number of counties with missing suicide rates, we instead report crude suicide rates, calculating rates for counties with 10–19 suicide deaths.

County‐level Veteran population projection data for 2017 were calculated by the National Center for Veterans Analysis & Statistics Veteran Population Projection Model 2016 (VetPop, 2016; U.S. Department of Veterans Affairs, Office of Enterprise Integration, & Data Governance and Analytics, 2017). VetPop2016 projects county‐level estimates for the Veteran population out to the Fiscal Year 2045 using the best available 2015 data from a range of sources collected for and reported by the Office of Enterprise Integration (Ahn, 2017).

### 2.3 Assumptions

To understand the applicability and appropriate interpretation of these planning maps, it is crucial to recognize that we (a) do not purport the three variables (county suicide rate, percent Veterans, and percent rural Veterans) to be statically predictive of one another, (b) are not suggesting that county suicide rates are necessarily correlated with Veteran suicide rates, and (c) do not assume that VHA‐enrolled Veterans are representative of all Veterans. While county Veteran‐specific suicide rates would have been preferable to county suicide rates, Veteran suicide rates are suppressed for the majority of rural counties due to small populations resulting counts of <10 for Veteran suicide deaths. Because Veteran suicide rates could not be computed for most rural areas, county adult suicide rates were chosen as a proxy. Rurality data from the VHA enrollee database were used because TWV is a VA ORH‐funded project, and majority rural VHA enrollment is an eligibility criterion for this project.

### 2.4 Approach

The datasets described above (excluding VA facility data) were used to create four mutually exclusive categories to represent eligibility for the TWV program and increasing levels of county need. The following three measures were used to determine eligibility and need: (a) rurality, considered met if the county had >50% of VHA enrollees residing in rural or very rural census tracts (an eligibility requirement for TWV), (b) suicide rate, considered met if the crude county rate was higher than the state median, and (c) Veteran population, considered met if the percent of Veterans residing in the county was higher than the state median. The latter two criteria were based off the state median instead of the national median so that counties with the highest need could be identified within each state. In order to reflect a more accurate county‐level median, counties with suppressed data (<10 suicide deaths) were given a value of 0 and included in calculating the median.

The four categories assigned to each county based on the three measures described above were (a) not eligible for TWV, (b) eligible for TWV and meets no other factors, (c) eligible and meets one factor, and (d) eligible and meets two factors. Choropleth maps using distinct shades of color to depict each county's category classification were created for each state using the eligibility and need factors as one layer with a second layer made up of VHA facilities. The eligibility factors choropleth layer was a monochromatic light‐to‐dark blue layer broken out by county with darker shades of blue applied to counties meeting more criteria.

All analyses were done in SAS version 9.4 (SAS Institute), and maps were created using ArcGIS Pro version 2.2.1 (Esri, 2018).

## 3 RESULTS

A total of 3113 counties were included in the analysis, with 2435 (78.2%) counties eligible to participate in TWV, as a majority of VHA enrollees (greater than 50%) in those counties live in rural areas. A total of 308 counties (9.89%) had suppressed suicide rates. The mean, standard deviation, and range for percent rural Veteran enrollees, percent Veteran population, and suicide rate can be found in Table 1. A list of median suicide rates and median Veteran populations for all states can be found in Supplementary Table S1.

**TABLE 1:** Descriptive statistics for county‐level variables

	*M* (*SD*)	Range
Percent rural VHA enrollees	80.37 (34.75)	0, 100
Percent veteran population	7.32 (2.13)	0.67, 23.05
Suicide rate per 100,000	18.15 (8.98)	0, 115.06

**FIGURE 1:**
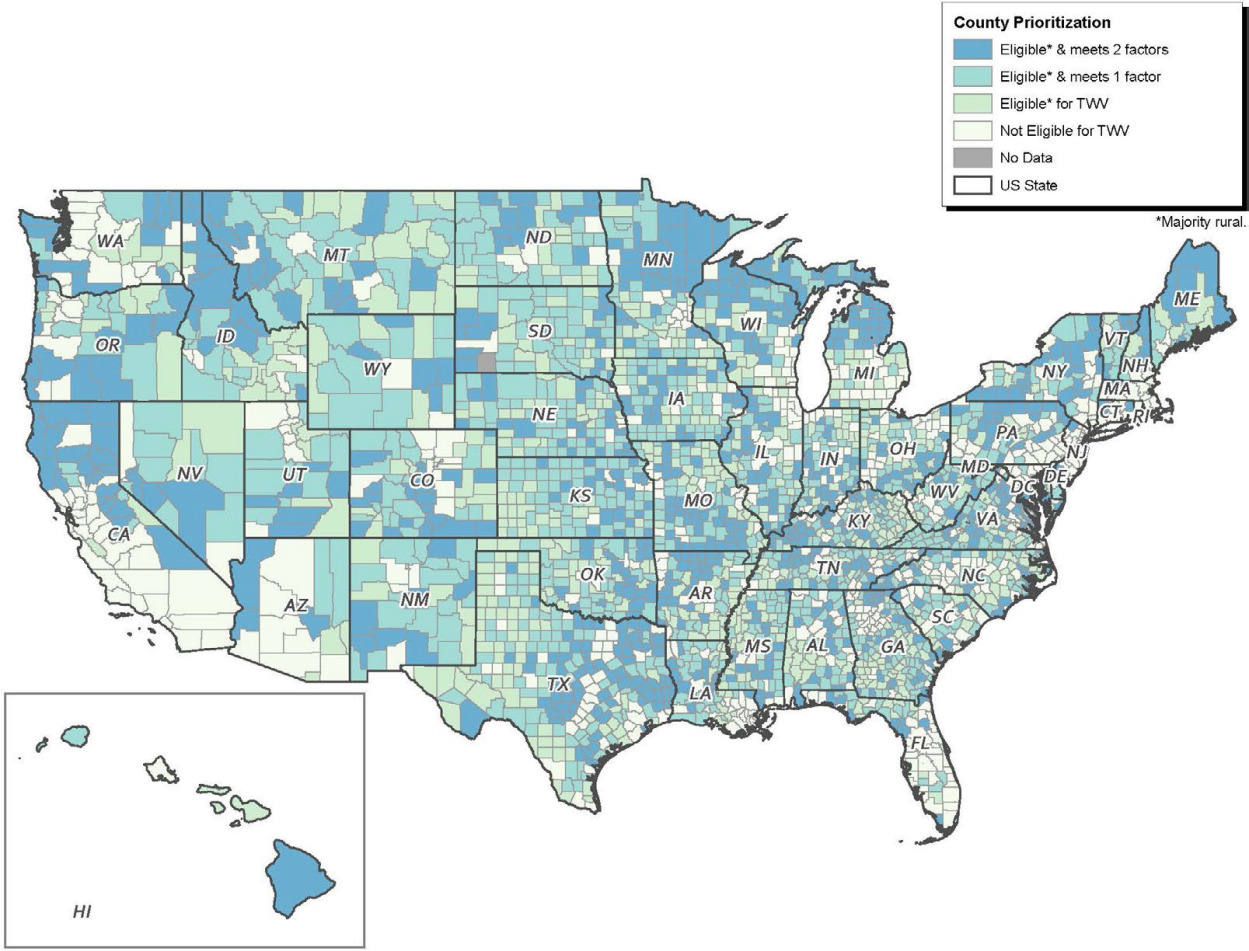
Together With Veterans national eligibility and need county map [Colour figure can be viewed at wileyonlinelibrary.com]

Figure 1 displays a national map of the U.S. counties demonstrating the distribution of counties deemed priority communities with darker colors indicating a higher level of need. Of the eligible counties, 643 (26.4% of eligible counties and 20.7% of all counties examined) met only rurality criteria (i.e., the proportion of Veterans living in the county and crude suicide rate for the county was not greater than the state median). There were 989 counties (40.6% of eligible counties and 31.8% of all counties analyzed), which were eligible for TWV and met one additional criterion (i.e., either the proportion of Veteran population was greater than the state median *or* the crude suicide rate for the county was greater than the state median). Eight hundred three counties (33.0% of eligible counties and 25.8% of all counties considered) fell into the highest need category (i.e., eligible for TWV, proportion of Veteran population, and crude suicide rate were both greater than state median). Washington D.C. and all five Rhode Island counties did not meet rurality criteria.

**FIGURE 2:**
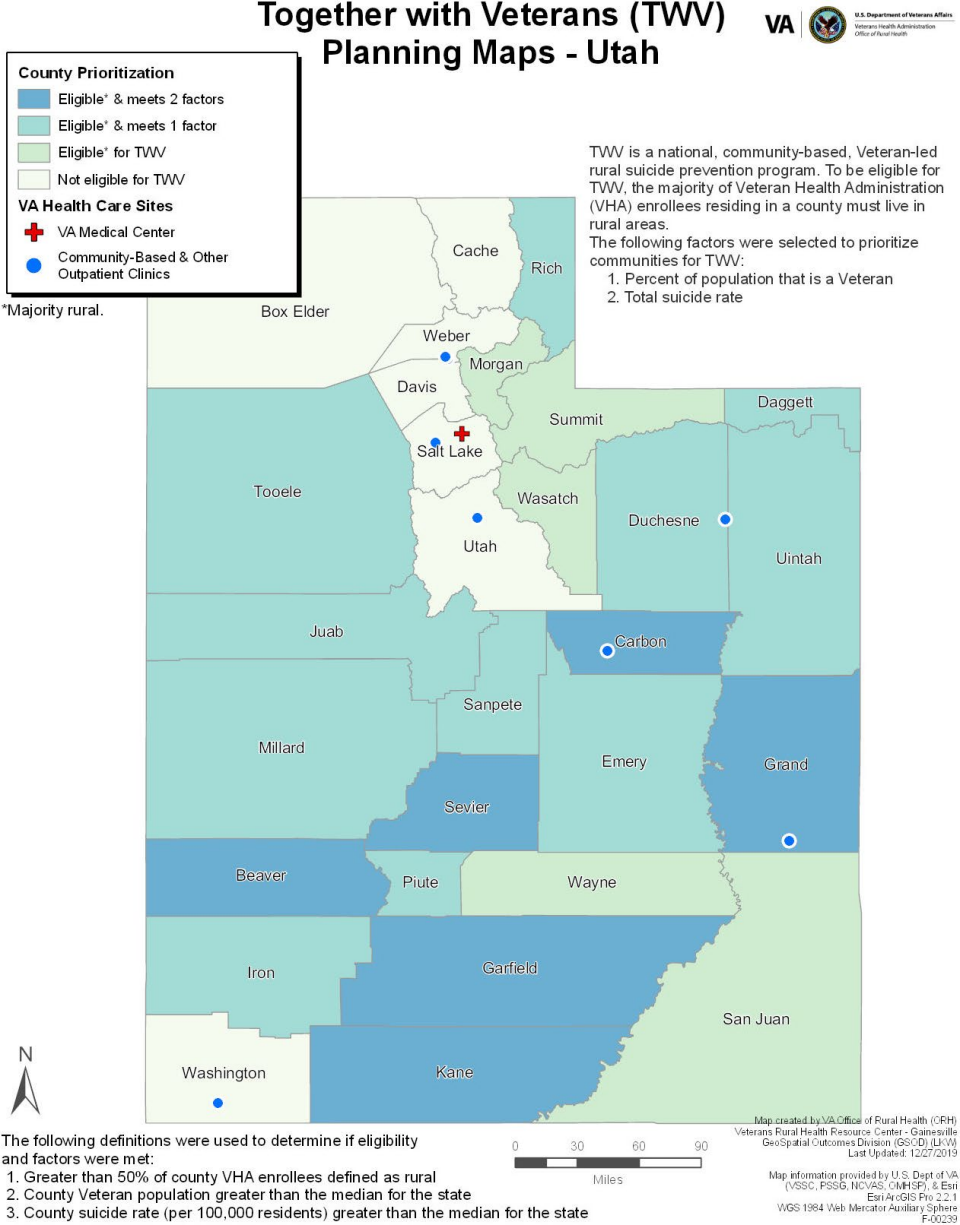
Together With Veterans final eligibility and need county map for Utah [Colour figure can be viewed at wileyonlinelibrary.com]

A map for each state was created with descriptive information to provide to partners and stakeholders as a stand‐alone document. Also included with each state map (not shown) was a table with county‐specific values for each measure and whether that value met each criterion. The map created for Utah is included as an example of the final product (Figure 2). In Utah, all but seven counties were eligible for the TWV program (i.e., greater than 50% of Veteran enrollees are rural). There were six counties (Beaver, Carbon, Garfield, Grand, Kane, and Sevier) that were eligible for the TWV program and met both additional factors (i.e., percent Veteran population and suicide rate higher than the state median) and should be considered first for the program. The VA Medical Center and CBOCs are concentrated mostly in the northern half of the state.

Table 2 provides recommendations for creating and using planning maps in a public health initiative. We encourage public health professionals to take several considerations into account for both the creation and use of the maps as they apply to individual projects.

**TABLE 2:** Recommendations for creating and using planning maps in a public health initiative

Project phases	Steps for consideration	As applied to current project
Creation of maps	Identify the eligibility criteria (as applicable)	Identified % of VHA‐enrolled Veterans who live in rural locations as the criteria for area eligibility for a rural health initiative
	Incorporate data for the target population	Target population is Veterans, and the data element is whether county Veteran population is over or under the state median
	Incorporate data for target health outcome	Target health outcome is suicide rates, and the data element is whether the county adult suicide rate is over or under the state median
	Treat maps as a discussion tool	Maps provided to generate discussion with state leaders to identify rural areas with need and capacity, and not inadvertently exclude a community identified locally as a priority for Veteran suicide prevention
Use of maps	Collaborate around interpretation of maps	Researchers/program analysts with knowledge of data and limitations engaged with key stakeholders with knowledge of the region
	Prioritize local context in decision‐making	Local contexts such as Veteran organizational capacity, available partnerships, presence or lack of existing suicide prevention efforts were used to identify communities where the program could succeed and not be redundant

## 4 DISCUSSION

Using available county‐level VA and CDC data on Veteran populations and suicide rates, we created 49 state maps to facilitate community selection for participation in the Together With Veterans program. The goal of TWV is to implement evidence‐based suicide prevention best practices (e.g., promote lethal means safety and improve access to crisis and support services) in rural communities while taking a Veteran‐driven, collaborative, and community‐centered approach (Monteith et al., 2020). The product we developed enables stakeholders involved in TWV community selection to glance at a map and quickly glean which counties in the state may have the highest need for and will likely benefit most from the program. By distilling and simplifying a crosswalk of multiple intersecting datasets, the maps provide a highly visual, readily comprehensible, starting point for suicide prevention planners to identify areas of greatest need. Notably, the maps did not exclude counties that only meet the TWV rurality requirement and not the other two factors. This was done to allow consideration of other important community factors that may make certain “lower need” counties particularly good candidates for the TWV program (e.g., demonstrating a high level of motivation and capacity to participate in the program). Additionally, this ensured that counties were not excluded for having a suppressed suicide rate, an important consideration in rural counties with lower populations that might still have high suicide risk and should be considered.

Given the limited resources of public health initiatives such as TWV, it is important to focus available resources on populations with the most need. As Hillier (2007) explains, when GIS is used in needs assessments, it can expose gaps across varying types of variables critical for promoting social justice and addressing the needs of more vulnerable populations. GIS can also improve the efficiency of programs or expand and increase access to services. Including locations of VA Medical Centers and CBOCs on the maps allows stakeholders to consider the ways in which these facilities can support suicide prevention in communities, given that mental health services have been a uniform requirement at these health centers since 2008 (U.S. Department of Veterans Affairs & VHA Mental Health Services, 2008).

We believe that targeting community partners for the TWV program by focusing on our specific variables of interest can increase the impact of our intervention. Additionally, we propose that our process can be applied to other public health issues and interventions, and provide Table 2 for professionals to reference in the creation and use of their own unique planning maps. While our process can be replicated to some extent for other public health initiatives, it is critical to understand that every type and source of data has unique strengths and limitations which may drastically change the applicability and interpretation of the end product.

### 4.1 Limitations

While GIS is dynamic in the sense that it can be changed and updated, the static maps used as part of this project do not display data in real time and are not automatically updated. As new data become available, the maps will be inaccurate if not updated regularly. The current maps only include data up to 2017 as this was the most recent CDC suicide data available. As a result, the data reflected in the maps used at the time of this paper could not utilize the latest available data for some areas and may reflect an inaccurate estimate of current need especially if suicide rates continue to rise (Kegler et al., 2017).

The use of three separate databases and use of a “proxy” variable for Veteran suicide rate (county adult suicide rate was used instead of Veteran suicide rate) limit the interpretations that can be drawn from the maps. The planning maps produced must be regarded as tools to aid in decision‐making and not as comprehensive tools to understand or predict county suicide risk. Understanding the background and context of these maps as an answer to community stakeholders’ requests and acknowledging they must be used alongside insights from local stakeholders is necessary to ensure appropriate use.

Though attempts were made to limit suppression by including 12 years of data and using crude rates instead of age‐adjusted, 9.89% of the counties were still suppressed as a result of low suicide counts. Due to this suppression, there is a possibility that counties that should be categorized as high need are not captured as such. We created four levels of need in our maps to ensure that counties with a suppressed suicide rate could still show up as higher need if there was a large enough Veteran population to account for this concern. Using crude instead of age‐adjusted rates when populations have varying age structures limits the ability to make meaningful comparisons across them (Centers for Disease Control & Prevention, 2019, 2017). The issue of suppression is another reason why, as stated in Table 2, “information on the local context is critical to mitigating limitations in the data.” Local and state stakeholders are key to interpreting the maps to ensure they are not used out of context.

Multiple definitions of rurality exist at the state and federal level, so there are inherent limitations in any project that uses a single rurality definition (Rural Health Information Hub, 2019). Since this project relied upon and required the use of Veteran enrollee data provided by VA, which uses a single rurality definition, using multiple definitions was not feasible for this project. There is also a limitation with using county‐level designations of rurality as opposed to smaller units like census tracts. Using county‐level rurality data can exclude rural communities that are located inside counties designated as urban (e.g., many of Arizona's counties are geographically very large and encompass both urban and highly rural census tracts) (Federal Office of Rural Health Policy, 2018). Therefore, when considering rural‐focused interventions, it can be important to consider the sub‐county level, though the existing data for our variables of interest were not available at that geographic level. The choropleth maps, therefore, are best served as a source of dialog to quickly identify areas of need and discuss best sites to launch the program.

### 4.2 Future directions

The current project relies primarily upon existing suicide risk (county suicide rate) and relevant community factors (percent Veterans and percent rural VHA enrollees) to prioritize counties for participation in a rural Veteran suicide prevention program. Since Alaska was excluded from analyses due to an inequivalent county classification system and change of census boundaries per the 2010 Census, we have reached out to the state and are working to identify a proper substate classification system that would allow us to create an appropriate planning map.

Future efforts should focus on expanding the use GIS and spatially referenced data to identify risk and hot spots for Veteran suicide for TWV and other VA programs. For instance, including other information such as distance or travel time from VA hospitals and clinics could add another layer of useful information for prioritizing counties for TWV since VA suicide prevention teams have limited outreach and need to rely more on local non‐VA partners, a strength of TWV. Additional steps may be to account for key social determinants of health that have a known association with suicide (e.g., age, unemployment, social integration, or substance use; Jalles & Andresen, 2015). Future projects would likely also benefit from including community resources such as Veteran‐related or faith‐based organizations, mental health facilities, crisis centers, other healthcare services, and interorganizational social networks for suicide prevention to further inform community environments.

In conclusion, it is critical that researchers and those residing outside of communities of interest do not view GIS as a stand‐alone tool for understanding communities, but as part of a set of tools. Additional tools might focus on evaluating community and organizational readiness, capacity for program sustainability, economic impact, and the impact of using different definitions of rurality (Rural Health Information Hub, 2020). The GIS maps developed for use in planning the dissemination of TWV are just that, a tool among others that can help healthcare systems scale up suicide prevention activities in a strategic fashion. By synthesizing complex and intersecting data, GIS can support effective scale up of public health suicide prevention programs.

## 6 DISCLAIMERS

The contents do not represent the views of the U.S. Department of Veterans Affairs or the United States Government.

## Supporting information

Table S1
