# Supplementary material for: Reducing rural veteran suicides: Navigating geospatial and community contexts for scaling up a national Veterans Affairs program
Source: Suicide Life Threat Behav. 2021 Apr 20;51(2):344–51. doi: 10.1111/sltb.12710 (PMC8252578; doi:10.1111/sltb.12710)
Supplement: Supplementary file 1 — Table S1 [file SLTB-51-344-s001.docx]

| Supplemental Table 1  *State Medians for Suicide Rate and Percentage of Population that is* *Veterans* | | |
| --- | --- | --- |
| State | Median Suicide Rate | Median Veteran Population |
| Alabama | 18.4 | 7.1% |
| Arizona | 25.4 | 8.0% |
| Arkansas | 21.3 | 7.4% |
| California | 17.0 | 5.9% |
| Colorado | 23.5 | 7.0% |
| Connecticut | 13.8 | 6.3% |
| Delaware | 15.9 | 9.2% |
| District of Columbia | 7.3 | 4.0% |
| Florida | 21.4 | 9.0% |
| Georgia | 17.0 | 6.8% |
| Hawaii | 20.7 | 6.1% |
| Idaho | 24.9 | 6.8% |
| Illinois | 15.4 | 7.4% |
| Indiana | 18.2 | 7.0% |
| Iowa | 15.8 | 7.3% |
| Kansas | 18.4 | 6.9% |
| Kentucky | 21.1 | 6.4% |
| Louisiana | 16.3 | 5.7% |
| Maine | 20.1 | 9.0% |
| Maryland | 14.7 | 7.4% |
| Massachusetts | 12.0 | 5.4% |
| Michigan | 19.0 | 7.8% |
| Minnesota | 16.2 | 7.1% |
| Mississippi | 16.2 | 5.6% |
| Missouri | 20.5 | 7.8% |
| Montana | 27.5 | 8.7% |
| Nebraska | 11.1 | 7.7% |
| Nevada | 34.1 | 9.3% |
| New Hampshire | 19.5 | 8.3% |
| New Jersey | 10.6 | 4.6% |
| New Mexico | 28.9 | 7.7% |
| New York | 14.0 | 7.1% |
| North Carolina | 17.7 | 6.8% |
| North Dakota | * | 7.0% |
| Ohio | 15.9 | 7.2% |
| Oklahoma | 23.2 | 7.5% |
| Oregon | 23.7 | 9.3% |
| Pennsylvania | 18.0 | 7.9% |
| Rhode Island | 13.9 | 7.1% |
| South Carolina | 17.4 | 7.3% |
| South Dakota | 10.6 | 6.9% |
| Tennessee | 23.0 | 7.2% |
| Texas | 17.4 | 6.3% |
| Utah | 26.2 | 4.8% |
| Vermont | 22.4 | 7.6% |
| Virginia | 19.1 | 7.9% |
| Washington | 21.2 | 9.0% |
| West Virginia | 21.2 | 8.0% |
| Wisconsin | 18.6 | 7.2% |
| Wyoming | 31.3 | 7.8% |

*29 of 53 counties have zero or suppressed suicide rates
